# How to behave when marooned: the behavioural component of the island syndrome remains underexplored

**DOI:** 10.1098/rsbl.2022.0030

**Published:** 2022-04-20

**Authors:** Ioanna Gavriilidi, Gilles De Meester, Raoul Van Damme, Simon Baeckens

**Affiliations:** ^1^ Functional Morphology Lab, Department of Biology, University of Antwerp, Wilrijk, Belgium; ^2^ Evolution and Optics of Nanostructures Lab, Department of Biology, Ghent University, Ghent, Belgium; ^3^ Section of Zoology and Marine Biology, Department of Biology, National and Kapodistrian University of Athens, Greece

**Keywords:** animal behaviour, behavioural syndrome, cognition, island evolution, personality

## Abstract

Animals on islands typically depart from their mainland relatives in assorted aspects of their biology. Because they seem to occur in concert, and to some extent evolve convergently in disparate taxa, these changes are referred to as the ‘island syndrome’. While morphological, physiological and life-history components of the island syndrome have received considerable attention, much less is known about how insularity affects behaviour. In this paper, we argue why changes in personality traits and cognitive abilities can be expected to form part of the island syndrome. We provide an overview of studies that have compared personality traits and cognitive abilities between island and mainland populations, or among islands. Overall, the pickings are remarkably slim. There is evidence that animals on islands tend to be bolder than on the mainland, but effects on other personality traits go either way. The evidence for effects of insularity on cognitive abilities or style is highly circumstantial and very mixed. Finally, we consider the ecological drivers that may induce such changes, and the mechanisms through which they might occur. We conclude that our knowledge of the behavioural and cognitive responses to island environments remains limited, and we encourage behavioural biologists to make more use of these ‘natural laboratories for evolution’.

## 1. Introduction

Animals and plants inhabiting islands tend to deviate from their mainland relatives in multiple aspects of their morphology, physiology, behaviour and life history; a pattern referred to as the ‘island syndrome’ [1–3]. A large body of literature on a variety of animal taxa documents how island populations stand out in body size and shape, colouration, locomotor abilities, diet, niche width, fecundity and lifespan, for instance (reviews in [2–7]). Curiously, far fewer studies have examined behavioural adaptations in island dwellers. Apart from the often documented phenomenon of ‘island tameness’ (i.e. insular prey species often fail to recognize or respond adequately to predators; [8,9]) and peculiarities of island birdsongs [10–12], whether and how insularity affects other behavioural aspects of animals has received far less attention. This is unfortunate, because behaviour is such an important part of the phenotype, and because many of the drivers of morphological, physiological and life-history evolution on islands are also likely to impinge on behavioural characteristics [13].

The island environment differs in many aspects from that of the mainland. Islands typically accommodate fewer species than mainland habitats of the same surface area [4,5,14]. The dearth of competitors, and especially of large predators, instigates ‘ecological release’ [15–17] which allows many insular prey species to reach much higher densities than on the mainland [18–20]. On the other hand, for many animals, dietary resources are often considered to be poor or more variable on islands compared to the mainland ([21–24], but see [25]). These environmental changes (i.e. poor or variable dietary resources, reduced predation pressure and interspecific competition, and increased intraspecific competition) are deemed responsible for many elements of the island syndrome described, although the mechanisms through which they exert their effects on animal phenotypes often remain elusive. Another line of research highlights the importance of environmental unpredictability on islands, and particularly on small islets, for the emergence of island syndromes [26,27]. We expect that the particularities of insular environments will likewise prompt adaptations in the behaviour of island dwellers. In this paper, we review the empirical evidence for a behavioural component of the island syndrome, focusing on two interrelated [28] behavioural domains: animal personality and cognition. We also consider the mechanisms and ecological drivers that could produce such changes.

Animal personality research has revealed, at least to some extent, repeatable and heritable inter-individual variation [29,30] in the way that animals behave in response to various external stimuli. Individual animals thus belong to one of several possible and coexisting ‘behavioural types’—groups that will respond in a distinctive, predictable way to challenges, even under different contexts. More specifically, individuals differ consistently in ‘personality traits’ such as boldness, aggression, activity, exploration and sociability (reviewed in [31]). Often, these personality traits intercorrelate, giving rise to a ‘behavioural syndrome’ [32]. Because island and mainland habitats differ in the opportunities and challenges they offer to their inhabitants, we can expect changes in the relative frequency of behavioural types or in the way that personality traits interconnect.

Cognition is the collective of neural processes responsible for the acquisition, retention and use of information [33] and, as such, a key determinant of fitness in vertebrate animals (e.g. [34–37]). Individuals differ in their cognitive abilities, but also in cognitive styles (e.g. learning speed versus accuracy [32]). Relatively little is known about the repeatability of cognitive testing in time, or across context [38]; or about how different cognitive scores interrelate [39]. In humans, scores on different cognitive tests tend to correlate positively, lending credibility to the concept of ‘general intelligence’ (the so-called ‘g-factor’ [40,41]). Similar covariation has been described for a handful of other mammals (e.g. [42]) and birds (e.g. [43]), but overall the evidence for a g-factor remains scarce [39,44]. Again, given how islands deviate from mainland habitats, different levels, types or sets of cognitive skills may be favoured in both types of environments.

## 2. The current evidence for a behavioural component to the island syndrome

Table 1 reviews the evidence that animals from islands differ from their mainland counterparts in aspects of their personality and cognition. Overall, few studies have thoroughly investigated the effect of insularity on personality. Careful analyses of animal temperament should consider whether individual behavioural scores (that are believed to reflect aspects of personality) are repeatable over time and over different contexts and whether and how these aspects covary to produce behavioural syndromes [32]. Inquiries into the evolution of insular personality will also require information on the heritability of such scores. Few of the studies in table 1 fulfil these conditions. Below we review behavioural studies that either measure personality traits (i.e. test for repeatability [105]) or behavioural changes (i.e. do not test for repeatability) that could indicate differences in personality.

**Table 1.** Studies that have compared behavioural, personality or cognitive (including brain morphology studies) traits between island and mainland, or among-island populations/species. The ‘driver’ and ‘mechanism’ are those suggested (but not necessarily demonstrated) by the authors. FID stands for flight initiation distance. The ‘type of analysis’ also indicates whether the study includes repeated within-individual measurements (personality variation). References to these studies were found by feeding the Web of Science and Google Scholar search machines with a combination of behavioural keywords (e.g. ‘personality’, ‘cognition’, ‘bold*’, ‘aggression’, ‘explorati*’) with terms referring to island environments (e.g. island, insular*, archipel*). We also carefully checked the reference lists of all papers for additional sources.

study species	trait	island	mainland	driver	mechanism	type of analysis	reference
personality							
eastern chipmunk (*Tamias striatus*)	island chipmunks are less vigilant, but both island and mainland specimens adjust vigilance to microhabitat structure	Beaver Island, Michigan, USA	two sites on mainland Michigan, USA	lower predation pressure on island (checked with camera traps)	unknown	field experiments; personality variation measured	[45]
14 species of macropodid marsupials	animals on islands are less wary and allocate more time to foraging	satellite islands of Australia	Australia	predation pressure	acknowledges possibilities of plasticity and selection	field observations; comparative analysis	[46]
reindeer (*Rangifer tarandus*)	island reindeer are more vigilant on the island, but response distances are the same	Edgeøya, Norway	four sites on Spitsbergen, Norway	possibly higher predation (by polar bears) on island	response distance believed to be ‘hard-wired'	field observations	[47]
bull-headed shrike (*Lanius bucephalus*)	longer FID in island shrikes	Kikaijima, Minami-daitojima and Nakanoshima Islands, Japan	three sites on main Japanse Islands	risk of predation (by rats) higher on islands	genetic change, plasticity and dispersal-related selection are considered	field observations	[48]
11 species of Falkland Island birds	FID is lower on island than on mainland	Falkland Islands	mainland Argentina	absence of terrestrial predators	probably innate, evolutionarily acquired, but habituation also deemed possible	field observations; comparative analysis	[49]
California quail (*Callipepla californica*)	FID is similar on mainland and island, but starting distance is smaller on islands	Santa Catalina Island, USA	California, USA	some predators lacking on island	assumed genetic (antipredator genes)	field observations	[50]
orange-throated whiptail (*Aspidoscelis hyperythra*)	island lizards are more difficult to catch than mainland lizards	seven Gulf of California islands	Baja California	predation pressure, human collection for pet trade	reasoned to be genetically based, implying rapid evolution of antipredator behaviour	field observations	[51]
Galápagos marine iguana (*Amblyrhynchus cristatus*)	lizards on predator-free islet have shorter FIDs; although FID increases with experience, it remains insufficient to avoid predation	Caamaño Islet, Galápagos, Ecuador	St Cruz and San Cristobal Island, Galápagos, Ecuador	predation pressure	release from predation narrowed the reaction norm for FID	field observations	[52]
spiny-tailed iguana (*Ctenosaura hemilopha*)	island lizards behave less wary and have shorter FIDs than mainland lizards	Cerralvo Island, Gulf of California	Mexico	low predation pressure on the island	no hints	field observations	[53]
lava lizards (eight populations of three *Tropidurus* spp.)	lizards on islands with introduced cats have higher FID	eight islands of the Galapagos	no mainland population	presence of exotic predators (cats)	could be inherited, or learned	field observations; comparative analysis	[54]
Ibiza wall lizard (*Podarcis pityusensis*)	FID and distance fled is greater on islets with higher predation pressure	seven islets around Ibiza and Formentera, Spain	no mainland population	predation pressure as estimated by number and kind of predators, incl. humans	unknown, both phenotypic plasticity and evolutionary processes deemed possible	field observations; comparative analysis	[55]
Lilford's wall lizard (*Podarcis lilfordi*)	FID, distance fled, hiding time and probability to enter refuge are lower on islet with less predators	islets of Rei and Aire, Menorca, Spain	no mainland population	predation pressure	natural selection	field observations	[56]
Italian wall lizard (*Podarcis siculus*)	FID is shorter on island with lower predation pressure	comparison between two islands in the Adriatic Sea	no mainland population	predation pressure, through number of predators and habitat structure	unknown	field observations	[57]
66 lizard species	FID decreases as distance to mainland increases	islands in the Atlantic and Pacific Oceans; Caribbean and Mediterranean Seas	five continents	predation pressure is lower on islands; reduced food availability may shorten FID to save energy	probably genetic changes, but tameness might be learned every generation	field observations; comparative analysis	[9]
Aegean wall lizard (*Podarcis erhardii*)	lizards from islets have shorter FID and act bolder than lizards from main island	four satellite islands of Naxos, Greece	18 populations on Naxos, Greece	cat predation	phenotypic plasticity; which is also maintained on islets	field observations; laboratory experiments	[58]
38 populations of Aegean wall lizard (*Podarcis erhardii*)	lizards from smaller and more isolated islets have shorter FIDs	37 Cycladic islands, Greece	mainland Greece	predation	natural selection	field observations	[59]
Lilford's wall lizard (*Podarcis lilfordi*)	FID and distance fled is not correlated with predation pressure	nine islets around Menorca and Mallorca, Spain	no mainland population	predation pressure as estimated by number and kind of predators, incl. humans	unknown, both phenotypic plasticity and evolutionary processes deemed possible	field observations; comparative analysis	[55]
Dalmatian wall lizard (*Podarcis melisellensis*)	islet lizards are bolder, less wary but not less neophobic than island lizards	three small islets out of the coast of Vis, Croatia	Vis, Croatia	food availability, predation pressure smaller on islets	selection, plasticity or non-random gene flow are suggested	field experiments	[60]
northern quolls (*Dasyurus hallucatus*)	animals on island behave less wary than mainland animals	Astell Island, a satellite island of Australia	Australia	absence of predation on island	natural selection	laboratory experiments	[61]
common frog (*Rana temporaria*)	enhanced boldness and exploration in island tadpoles and froglets	four islands in the Gulf of Bothnia, Sweden	Sweden	dispersal propensity; unstable conditions on islands (pond drying); reduced predation on islands	founder effects caused by environmental filtering or differential natural selection	laboratory experiments with wild-caught specimen; personality variation and behavioural syndrome measured	[62]
house mice (*Mus musculus domesticus*)	enhanced boldness and exploration on island	Gough Island, Tristan da Cunha	Maryland, USA	novel food source (sea birds), loss of predatory danger, removal of human commensals, variable food availability	genetic change	common garden experiment with F1-offspring	[63]
61 species of parrots (Psittacidae)	island species explore novel objects faster and longer than mainland species; island species are not less neophobic than mainland species	several, not specified	several sites, not specified	reduced predation pressure and higher risk of food shortage on islands	assumed genetic	comparative analysis; behavioural syndrome across species tested, but not at the individual level, or between island and mainland species	[64]
island scrub jay (*Aphelocoma insularis*) versus California scrub jay (*A. californica*)	island birds were more explorative than mainland birds	Santa Cruz Island, California, USA	mainland California, USA	reduced predation, more frequent food shortage on island	unknown	field observations/experiments	[65]
brown anole (*Anolis sagrei*)	lizards on islands with introduced predators are less explorative	eight small islands in the Caribbean, on four of which predatory lizards were introduced	no mainland population	presence of introduced predators (lizards)	natural selection	fitness gradient analysis; personality variation measured	[66]
red-backed vole (*Myodes gapperi*)	island voles are less aggressive than mainland voles; no difference in exploration	10 islands in the Winnipeg River, Ontario, Canada	six sites on mainland Ontario	relaxed predation, higher population density	dispersal-related, ecological and evolutionary mechanisms all considered	laboratory experiment; comparative analysis	[67]
deer mouse (*Peromyscus maniculatus*)	wild-caught island mice are less aggressive, but difference disappears in subsequent generations	Saturna Island, Pender Island, Canada	British Columbia, Canada	population density thought to reduce aggressiveness	phenotypic plasticity	laboratory experiment with wild-caught specimen and their offspring; crossings	[68]
deer mouse (*Peromyscus maniculatus*)	island mice do not show aggressive behaviour towards juveniles; some mainland mice behave aggressively towards non-kin	Saturna Island, Canada	British Columbia, Canada	reduced intraspecific competition due to high food supply on island	unknown	laboratory test on P (interact with F1)	[69]
common shrew (*Sorex araneus*)	island and mainland shrews equally aggressive	four islands in the Baltic Sea	two sites on mainland Finland	island specimens often inbred		laboratory experiments with field-caught individuals	[70]
Skyros wall lizard (*Podarcis gaigeae*)	islet lizards more likely to attack juveniles and behave more aggressively to other adults	islet Diavates, Skyros archipelago, Greece	Skyros main island, Greece	food scarcity and high population size prompt for cannibalism	Unknown	laboratory experiments with field-caught individuals	[71]
Italian wall lizard (*Podarcis siculus*)	island lizards are more aggressive than lizards on the mainland	Licosa Island, Italy	one mainland site (Punta Licosa), Italy	possibly low or fluctuating population density on island	unknown	field observations; laboratory experiments	[26]
tiger snake (*Notechis scutatus*)	adult snakes from predator-rich sites have more vigorous responses when handled, but neonatal behaviour is unrelated to predator species richness	eight islands around Australia	three sites, on mainland Australia and Tasmania	predation pressure, through number and type of predators	ontogenetic plasticity; experience, or genetically coded adjustment of behaviour to ontogenetically variable traits	observations on freshly caught individuals	[72,73]
common garter snake (*Thamnophis sirtalis*)	adult but not neonate snakes from the mainland behave more aggressively towards experimentor than island snakes	Beaver Archipelago, USA	Michigan, USA	fewer predators on island	both innate and environmental influences	laboratory behavioural observations	[74]
Pacific rattlesnake (*Crotalus oreganus*)	island snakes behave more aggressively towards humans	Santa Catalina Island, USA	mainland California, USA	island has fewer avian predators but perhaps more (introduced) mammalian predators	unknown	field observations	[75]
chuckwalla species (*Sauromalus* spp.)	island endemics are more sociable, less aggressive than mainland species	San Esteban Island and Angel Island, California, USA	mainland California, USA	dearth of predators, competitors, niche expansion, high but fluctuating food supply, high density on island	unknown	field observations	[76]
house mice (*Mus musculus domesticus*)	island mice do not show aggressive, defensive or cautious behaviour towards conspecifics	Isle of May, UK	Nottinghamshire, UK	interaction between resource distribution, habitat structure and predation risk	unknown	laboratory experiments on recently caught specimens	[77]
yellow-faced grass quit (*Tiaris olivacea*)	island birds are more territorial than mainland birds, which occur more often in flocks	Jamaica	Costa Rica	island density is lower	unknown	field observations	[78]
European earwig (*Forficula auricularia*)	island populations with high densities have larger proportion of macrolabic (fighter) morphs	35 British islands	11 mainland Britain sites	population density	intraspecific competition	laboratory experiments	[79]
human (*Homo sapiens*)	islanders exhibit greater animosity towards strangers and keep greater social distance	Croatian Islands	mainland Croatia	dangers associated with infectious disease		questionnaire	[80]
human (*Homo sapiens*)	islanders had higher levels of consciousness, emotional stability and lower levels of extraversion and openness; no difference in agreeableness	Giglio, Ponza and Ventotene, and seven Aeolian islands, Italy	three sites on mainland Italy	island is harsh, restricted environment with limited social environment	assumed adaptive (changes in same direction); elimination of well less adapted through mortality, assortative mating or emigration	questionnaire	[81–83]
meadow vole (*Microtus pennsylvanicus*) versus beach vole (*M. breweri*)	island species is more sociable	Muskeget Island, Massachusetts, USA	mainland Massachusetts, USA		differential dispersal of intolerant specimens	field observations	[84]
46 species of birds	birds on islands tend to flock less than birds on the mainland	22 different islands	22 mainland sites to match islands	predation pressure	random drift or active selection are suggested	comparative analysis	[85]
long-tailed macaque (*Macaca fascularis*)	smaller group sizes on island	Simeulue, Indonesia	Sumatra, Indonesia	felid predation	unknown	field observations	[86]
tammar wallaby (*Macropus eugenii*)	time allocation is dependent on group size on mainland and islands with reduced number of predators, but not on predator-free island	Garden Island, Kangaroo Island, Australia; Kawau Island, New Zealand	Western Australia	absence of predators	maintained by natural selection, but ‘priming agents’ may be required to develop antipredator behaviour	field observations	[46]
oriental fire-bellied toad (*Bombina orientalis*)	island toads have lower levels of activity	Jeju Island, South Korea	two sites on mainland South Korea	predation is higher on island	local selection, founder effects also considered possible	laboratory observations	[87]
clouded anole (*Anolis nebulosus*)	island anoles are more active	San Agustín, Mexico	mainland Mexico	less variable environmental conditions on island may allow better thermoregulation; higher predation on mainland		field observations	[88]
cognition							
Minatogawa man (*Homo sapiens*) versus modern Japanese and Pleistocene/Holocene *H. sapiens*	Pleistocene island dwellers had relatively small endocrania	Okinawa Island, Japan	mainland Japan	undernutrition	phenotypic plasticity; genetic adaptation	comparative analysis of brain size	[89]
Human (*Homo floresiensis* versus *Homo erectus*)	relative brain size is lower in island species	Flores, Indonesia		biotic interactions and resource use	natural selection for smaller brains (in addition to selection for smaller body size)	quantitative genetic modelling	[90]
mouse and dwarf lemurs (Cheirogaleidae)	disproportional reduction in brain size in this island clade	Madagascar		unpredictable food availability	natural selection	comparative analysis of brain size	[91]
Malagasy dwarf hippo (*Hippopotamus lemerlei*, *H. madagascariensis*) versus hippopotamus (*H. amphibius*)	relative brain size is lower in island species	Madagascar	mainland Africa	poor dietary resources on islands	natural selection for smaller brains (in addition to selection for smaller body size)	ontogenetic modelling	[92]
Siculo-Maltese dwarf elephant (*Palaeoloxodon falconeri*) versus *P. antiquus*	relative brain size is lower in island species	Malta	mainland Africa	poor dietary resources on islands	natural selection for smaller brains (in addition to selection for smaller body size)	ontogenetic modelling	[92]
Siculo-Maltese dwarf elephant (*Palaeoloxodon falconeri*) versus *P. antiquus*	dwarfed insular species has a high encephalization quotient	Sicily, Italy	mainland Europe		need to maintain the minimal functional volume of the brain when the size of the skull was drastically reduced	allometric analysis	[93]
Balearic Islands cave goat (*Myotragus* spp.) compared to 54 spp. of extant bovids	insular species have small brain and sense organs relative to body size	Balearic Islands, Spain	mainland Africa	absence of predators; overpopulation; limited energy availability	natural selection for smaller brains (in addition to selection for smaller body size).	scaling analysis	[94]
Cretan deer (*Candiacervus*), compared to extant deer (Cervidae)	insular dwarf deer have normal relative brain size	Crete, Greece		dearth of predators on islands		comparative analysis of brain size	[95]
Sardinian dhole (*Cynotherium sardous*), compared to two spp. of extant dog spp. (Canidae)	insular dwarf canid has normal relative brain size	Sardinia, Italy				comparative analysis of brain size	[96]
Minorcan giant rabbit (*Nuralagus rex*), compared to extant rabbit species (Leporidae)	Late Neogene insular giant had relatively small brain; especially sense-dependent areas are small	Minorca, Balearic Islands, Spain		absence of predators; limited energy availability		comparative analysis of brain size	[97]
426 mammalian species	no effect of insularity on relative brain size	islands worldwide	mainland sites throughout the world	poor dietary resources on islands	natural selection for smaller brains	comparative analysis of brain size	[98]
dodo (*Raphus cucullatus*) compared to nine spp. of pigeons (Columbiformes)	endocranial volume not smaller than expected from pigeon allometry	Mauritius				allometric analysis	[99]
Rodrigues Island giant owl *(Otus murivorus*), compared to 10 extant spp. of owls (Strigidae)	reduction of brain volume in extinct island endemic	Rodrigues, Mauritius	diverse	absence of predators, reduction of interspecific competition	brain expansion cannot follow pace of body size increase (evolutionary pace dissociation)	comparative analysis of brain size	[100]
Haast's eagle (*Harpagornis moorei*), compared to 35 spp. of eagles (Accipitridae)	island endemic had low endocranial volume for its body mass	New Zealand	diverse	absence of predators, competitors on island	mismatch between neural and somatic growth	comparative analysis of brain size	[101]
40 crow and raven species (*Corvus*)	brain size does not predict ability to colonize islands	islands worldwide	mainland sites throughout the world	islands are challenging environments, promoting enhanced cognition		comparative analysis of brain size	[102]
1900+ species of birds	insular species have larger brains	diverse	diverse	high environmental unpredictability across years	natural selection; high phenotypic plasticity may inhibit evolutionary change in some clades	comparative analysis of brain size	[103]
Deer mouse (*Peromyscus maniculatus*)	insular mice displayed shorter latencies to solve a Morris water-maze task	Moresby Island, Canada	British Columbia, Canada	differences in swimming abilities (rather than cognitive skills) between populations	unknown	laboratory observations	[104]

### (a) Boldness

Boldness, i.e. risk-taking behaviour in the presence of a predator threat, is expected to change in a way that matches predation risk on islands. The few studies focusing on the effect of insularity on boldness have found island common frogs (*Rana temporaria*) [62] and house mice (*Mus musculus domesticus*) [63] to be bolder than their mainland counterparts. Perhaps inspired by early (mostly anecdotal) accounts of island tameness (e.g. [8,106,107]), most studies investigating the effect of insularity on animal behaviour have focused on vigilance and risk-taking, common proxies for boldness. The vast majority of these studies (table 1) confirmed that island animals, especially those living on remote, predator-poor islands, tend to be less vigilant than their relatives inhabiting the mainland or less remote, predator-ridden islands [9,108]. Reindeer (*Rangifer tarandus*) on Norwegian Edgeøya [47], bull-headed shrikes (*Lanius bucephalus*) on Japanese Nansei islands [48] and whiptail lizards (*Aspidoscelis hyperythra*) on islands in the Gulf of California [51] are exceptions, but their relative shyness is attributed to unusually high densities of predators (polar bears, rats and human collectors, respectively) in their habitat. However, it should be noted that most of these studies rely on field observations of the flight initiation distance (FID), a proxy of boldness that is not without difficulties. FID is known to depend on a variety of factors related to the internal status of the animal (e.g. satiation [109]; reproductive status [110]; body condition [111]), and its environment (e.g. temperature [112]; substrate type [113]; levels of human exposure [114]; social context [115]; distance to safety [116])—all of which may differ consistently between island and mainland sites. In addition, FID measures boldness as an animal's response in a single context (a human approaching), without testing whether inter-individual variation is consistent and repeatable across time and context (i.e. personality *sensu stricto* [31]). Therefore, future studies should test the boldness of island and mainland conspecifics in multiple, controlled contexts. This could be done for instance by observing the animals' behaviour (e.g. avoidance and inspection of predator models, startles, latency to resume feeding) when confronted with varied threat stimuli (e.g. predator cues, loud noise and novel objects) (see [31] for a review of techniques; and [60–63] for examples in island–mainland comparison).

Increased boldness on islands is typically attributed to reduced predation pressure, although quantitative or even qualitative estimates thereof are seldom presented. The (often implicit) rationale is that bold behaviour is less penalized in predator-poor environments and might even be favoured for its paybacks in a non-predatory context [117,118]. It should be noted that while many (oceanic) islands are indeed devoid of predators, others hold thriving predator populations (e.g. [119–121]). Bold anti-predatory behaviour could also evolve as a behavioural mechanism to deter some of these predators (see [122] for a curious example in New Zealand parrots). Future research on this topic should be careful to match-up behavioural traits of island animals and the predators they were exposed to through evolutionary time.

The mechanisms through which the differences in vigilance and boldness arise remain unclear. Most authors consider both phenotypic plasticity (learning) and genetic adaptation, usually with a slight preference for the former. In a rare study tackling the question directly, Stratton *et al*. [63] found that differences in boldness between Gough Island and mainland US mice (*Mus musculus*) persisted in the F1 generation, offering support for genetic change [63].

### (b) Explorativeness

We found only seven studies that investigated the effect of insularity on animal explorativeness (i.e. behavioural response to novelty), and these produced mixed results (table 1). Common frogs (*Rana temporaria*) on Bothnian islands [62] and giant house mice (*M. musculus*) on Gough Island [63] are more explorative than their conspecifics on the mainland; survival of brown anoles (*Anolis sagrei*) that were more inquisitive was lower on islets with predators than on predator-free islets [66], and among parrots (Psittacidae) [64] island species tend to be more neophilic and explorative. A study on the exploratory and neophilic behaviour of scrub jays (*Aphelocoma coerulescens*) [65] concords with these results. Most studies indicate that predation relaxation and unpredictable resource availability may be drivers of increased explorativeness on islands [123]. The mechanisms of change in explorativeness on the islands are less clear. Lapiedra *et al*. [66] provided evidence that the behavioural changes in brown anole (*A. sagrei*) populations following the introduction of a predator were due to natural selection [66]. Brodin *et al*. [62] argued how dispersal-related environmental filtering could be responsible for the higher explorativeness in island common frogs and tadpoles [62]. By contrast, deer mice (*Peromyscus maniculatus*) on islands in the Canadian Winnipeg River tended to be less explorative than mainland conspecifics, possibly reflecting differences between inland and oceanic island systems [67]. Camperio Ciani *et al*. showed that human inhabitants of the Egadi islands are less open to new experiences than their compatriots in mainland Italy, an observation they attribute to disproportionally high emigration of individuals with neophilic personalities [81,82]. They even identified a genetic polymorphism that could be associated with this ‘personality gene flow’ phenomenon [83].

### (c) Aggression

The empirical evidence on changes in aggression towards (but not necessarily limited to) conspecifics go either way. Reduced antagonism in island populations, as the result of high densities that make the monopolization of resources ineffective [124], was reported in five studies on a few rodent species (*P. maniculatus* [68,69], *Myodes gapperi* [67] and *M. musculus* [77]) and birds [11]. Interestingly, Baier & Hoekstra [68] found that the difference faded in the F1 generation, indicating an important role for phenotypic plasticity [68]. In reptiles, the results are less congruent. In tiger snakes (*Notechis scutatus*) [72,73] and common garter snakes (*Thamnophis sirtalis*) [74], adults (but not laboratory-raised juveniles) fit the pattern of reduced aggression on islands, suggesting that insular conditions induce a plastic response of soothing in these animals as well. However, Pacific rattlesnakes (*Crotalus oreganus*) from Santa Catalina Island behave more aggressively than mainland conspecifics; perhaps because of the high number of (recently introduced) terrestrial predators on the island [75]. Skyros wall lizards (*Podarcis geigeae*) on smaller islets attack conspecifics frequently, probably because food shortage forces them into cannibalism [71].

### (d) Sociability

The effects of insularity on sociability (i.e. the quality of being social) have hardly been studied. Older papers report that meadow voles (*Microtus breweri*) [84] and chuckwallas (*Sauromalus* spp.) [76] living on islands tend to be more sociable, than closely related species on the mainland. By contrast, yellow-faced grass quits (*Tiaris olivacea*) [78] and birds in general [85] are seen in flocks more often on the mainland than islands. Long-tailed macaque (*Macaca fascicularis*) group size tends to be larger on the mainland [86]. However, whether changes in group size actually reflect differences in sociability (a personality trait), requires further behavioural testing. Predation risk, population density, food availability and habitat structure are among the ecological factors suspected to influence sociality (i.e. the tendency to associate with other individuals) [85,86,125,126], but the mechanisms remain utterly unexplored. Work on humans suggests that islanders have lower levels of extraversion and openness, and exhibit greater animosity towards strangers, keeping for instance greater interpersonal distance [80–82].

### (e) Activity

We found very little information on differences in the level of activity (an animals' tendency to engage in physically demanding behaviours) displayed by island and mainland animals. Clouded anole lizards (*Anolis nebulosus*) are more active on islands [88], while insular oriental fire-bellied toads (*Bombina orientalis*) exhibit lower activity levels compared to mainland conspecifics [87]. Predation pressure, population density, resource availability and thermal conditions are considered to shape activity [87,88,127]. We did not find any studies concerning the mechanisms of activity level changes. Therefore, the dearth of information precludes any form of generalization. This is a pity, because there is currently great interest in the causes and consequences of variation in the amount of activity that animals display (see [128]), and mainland–island comparisons might have provided a nice insight into the matter.

### (f) Cognition (with brain size as surrogate)

Studies on a variety of mammals and birds report a reduction in brain size on islands. However, interpretation and generalization of these results is problematic. First, most of these studies have been performed on fossil species and are therefore fraught with practical difficulties concerning the estimation of brain size and body size (see e.g. the disparate results for the Siculo-Maltese dwarf elephant (*Palaeoloxodon*) [92,93]). Second, no evidence for insular brain size reduction was found for other species, such as the Cretan deer (*Candiacervus* spp.) [95], the Sardinian dhole (*Cynotherium sardous*) [96], or even the long-thought-dim dodo (*Raphus cucullatus*) [99]. Third, recent comparative studies on extant mammals [98] and crows and ravens [102] found no effect of insularity on relative brain size. A meta-analysis of over 1900 species of birds even suggests a tendency towards larger brains in insular species. The authors argue that island species require larger brains to cope with the difficulties of having to exploit novel dietary resources and deal with high environmental stochasticity [103]. Fourth, and most importantly, it has become increasingly clear that brain size must be considered a shaky proxy for cognitive capacity (e.g. [129,130]).

### (g) Cognition (with behaviour as surrogate)

More direct evidence comes from a few studies that have compared aspects of cognitive capacity between island and mainland populations. Deer mice (*P. maniculatus*) from Morseby Island solved a water-maze task faster than conspecifics from mainland British Columbia, but this was attributed to differences in swimming rather than cognitive skills [104]. White-faced capuchins (*Cebus capucinus*) from a single island in Coiba National Park, Panama, engage in innovative tool use, a behaviour never observed in mainland conspecifics [131]. Tool use is also remarkably often reported in island birds (e.g. New Caledonian crows (*Corvus moneduloides*) [132], Hawaiian crows (*Corvus hawaiiensis*) [133], woodpecker finches (*Cactospiza pallida*) [134], Goffin's cockatoos (*Cacatua goffini*) [135]), but no one seems to have checked for a general association between tool use and insularity. On the whole, the number of studies examining the evolutionary fate of cognition and behavioural flexibility on islands is very limited.

## 3. Routes towards insular behaviour

**Figure 1.**
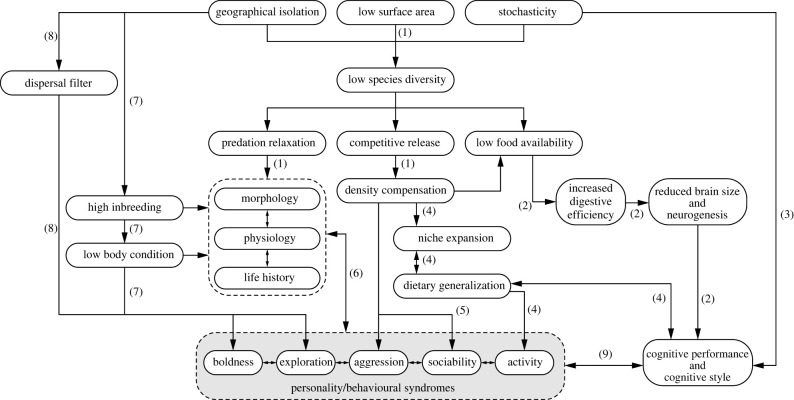
Putative relationships between island conditions, personality and cognition. Arrows with (1) represent ‘traditional’ pathways leading to the island syndrome. Pathway (2) echoes the ETH [136], predicting lower cognitive capacity in island populations. By contrast, (3) depicts the possibility that the unpredictable nature of the island environment selects for behavioural flexibility, requiring high cognitive capacity [123]. Route (4) represents a possible connection between niche expansion, dietary specialization, and aspects of personality and cognition (as proposed by [137,138]). Arrows with (5) indicate that high densities could lead to reduced territoriality and changes in how animals interact behaviourally (e.g. [77]). Arrow (6) summarizes the multiple connections between ‘traditional’ elements of the island syndrome and personality, e.g. through pleiotropic effects [139] or correlated selection (e.g. pace of life syndrome [140]). Pathway (7) concerns non-adaptive consequences of inbreeding on personality and cognition (e.g. [141]). Route (8) describes a possible role for selection on dispersal-related personality traits (e.g. [142]). Finally, the arrows labelled (9) summarize ideas on how personality and cognition might interact (e.g. [143]).

Below we review the multiple mechanisms that could produce such changes (also see figure 1). Because these routes are rarely documented in island–mainland comparisons, some of our examples come from other study systems. However, we try to explain why we think particular drivers and pathways seem likely to be especially relevant in an insular context.

### (a) Phenotypic changes

Personality traits can change within the lifetime of an individual, under the influence of environmental conditions experienced during (sometimes remarkably short) time windows during development [144–146]. For instance, smaller fathead minnows (*Pimephales promelas*) behave more boldly than larger conspecifics when raised in low-risk environments, but not when raised under high-perceived risk [147]. Jumping spiders (*Marpissa muscosa*) raised in socially enriched environments (i.e. raised in conspecific groups) demonstrated higher exploration later in life [148]. Hence, key components of insular environments such as reduced predation risk and increased population density could instigate changes in personality, even without genetic differentiation.

Cognitive capacity is notoriously plastic. In humans and traditional animal models, such as rats, pigs and guinea pigs, both intrauterine [149] and postnatal [150] undernutrition impedes neuronal and cognitive development. Effects have been observed in brain architecture, and on cognitive capacities such as spatial and reversal learning, memory and novelty seeking [149,150]. On the flip side, favourable developmental conditions are known to induce a ‘silver spoon’ effect, with positive effects on cognitive capacity. For instance, blue tits raised on taurine-enriched diets demonstrated improved memory and learning skills [151]. Islands tend to be relatively poor in dietary resources [21–24], and this may directly and negatively impact cognitive capacity. However, due to ecological release and niche enlargement, islands may sometimes provide more food per unit effort (e.g. [152]) and thus might constitute ‘silver spoon’ environments that boost cognitive development. Changes in foraging behaviour that accompany niche expansion may reverberate in personality [137] and cognitive [138] traits. Island dwellers may also experience different social contexts during ontogeny than their mainland relatives, as a consequence of high population density. In group-living animals, such as Australian magpies (*Cracticus tibicen*), growing up in a rich social environment boosts performance in a variety of cognitive tasks [37,153].

### (b) Genetic changes

Alternatively, since both personality and cognition traits display heritable variation [32,154], any evolutionary mechanism influencing allele frequencies may cause differences between island and mainland populations.

Genetic differences may arise by chance: through a founder effect (if allele frequencies of the colonizing propagule happen to differ from that of the mainland source population), or when random drift haphazardly alters the genetic constitution of the newly established island population [155,156]. Raffard *et al*. [157] found that the differentiation in boldness among 13 populations of a freshwater fish (the European minnow, *Phoxinus phoxinus*) could be attributed to random drift [157]. Other studies mention genetic drift as a potential cause of among-population variation in personality or cognitive traits, although often as a less glorious alternative to natural selection (e.g. [158]).

In the early stages after colonization, populations on oceanic islands may be prone to inbreeding [159], which typically results in increased homozygosity and decreased body condition [160,161]. If personality is condition-dependent (which seems plausible [162], but remains debated [141]), inbreeding may result in non-adaptive changes in average personality traits. In accordance, Verweij *et al*. [163] found negative associations between the level of inbreeding and personality traits such as novelty seeking and persistence in Finnish and Australian citizens [163]. In a rare study of the effects of inbreeding on animal personality, Müller & Juškauskas [164] found that inbred individuals of the leaf beetles (*Phaedon cochleariae*) behaved more boldly than outbred conspecifics [164]. Evidence that human cognitive abilities may suffer from inbreeding depression comes from genealogical studies on consanguineous marriages (e.g. [165]), including those of royal lineages (e.g. [166]). These traditional studies have recently been substantiated by genome-wide association studies describing negative associations between levels of inbreeding (homozygosity) and human intelligence [167–170], demonstrating that effects on cognition are not restricted to recent inbreeding events. Studies on the effect of inbreeding on animal cognitive capacity are extremely rare [171], but inbred lines of rats tend to exhibit cognitive deficits compared to outbred lines (e.g. [172]). In fruit flies (*Drosophila melanogaster*), Nepoux *et al*. [171] found a negative effect of severe inbreeding on aversive learning [171].

Differences in personality or cognitive traits between island and mainland populations could also arise through pleiotropic effects, i.e. when alleles that are selected because of their effect on unrelated morphological, physiological or life-history traits also happen to shape behaviour [139,173–175]. More concrete evidence for such piggyback riding of behavioural genes comes from artificial selection studies, in which selection for one trait has (often unexpected) effects on other characteristics. For instance, selection for high voluntary wheel-running activity in mice resulted in reduced aggressiveness towards conspecifics [176]. Selection for wheat digestibility in broiler chickens affected the birds' neophobia, sociability and explorativeness [177]. In *Drosophila*, selection for both nutritional stress resistance [178] and longevity [179] came at the cost of reduced learning ability. These studies have not worked with island populations, but high locomotory activity, increased digestive abilities, nutritional stress and longer lifespans have all been associated with insular conditions (e.g. [88,180,181]). As an example of how pleiotropic effects might come about at the molecular level, consider how the vertebrate melanocortin system affects a variety of traits, including colouration, immunity, energy expenditure and stress resistance, but also aggression and sexual activity [139]. In principle, altered background-matching requirements on islands might select for darker colouration by increasing the activity of melanocortin receptors, which would collaterally increase aggressiveness and sexual activity. Pleiotropic effects have been invoked to explain the multifaceted changes in island lizards [26], although in this study, the behavioural components (aggressiveness, voraciousness) were deemed the targets of selection, and the changes in colour a happy by-product.

Perhaps the most intuitive path towards genetic differentiation in personality or cognition between island and mainland populations is through natural selection. As recognized by studies of dispersal reduction ([182–184], but see [185,186]), island dwellers are likely to have been exposed to at least two successive bouts of selection during their evolutionary history: on their route to the island, and subsequently, when confronted with the new environment. The nature and even the direction of selection during these two stages may diverge strongly [187]. Trait values that facilitate dispersal to and colonization of islands may diverge from (or even oppose) those that benefit fitness once the population is established [188]. Which of the two selection bouts will be most reflected in the island population, will then depend on the time since colonization, and the plastic and/or evolutionary malleability of the trait concerned. Recent studies have documented instances of very rapid dispersal reduction in some insect and plant taxa (e.g. [185,189]), but there are also cases where dispersal capacity does not or only very slowly decreased post-colonization [190,191].

Dispersal barriers may act as a filter for or isolate island dwellers of certain personality and cognitive phenotypes. There is now ample evidence for personality-dependent dispersal (reviewed in [142,192]). Aggressiveness may influence dispersal either way. Antagonistic individuals may coerce more peaceful conspecifics to disperse [193,194], or move away themselves [195,196]. The relationship between sociality and dispersal tendency seems also taxon-specific (compare [197] with [198]) or density-dependent [199]. The relationship between dispersion and cognition has received little attention [200,201]. In theory, both positive and negative relationships could evolve: well-developed cognitive skills may help dispersers survive the perilous route towards new horizons; but individuals that invest heavily in cognition may be reluctant to disperse into unknown territories (e.g. [202]). Comparative research on wide a variety of animals suggests that cognitive abilities are a determinant of invasion success (reviewed in [203]), but these studies typically emphasize the role of cognition in coping with new challenges encountered in the invaded territories, rather than with the dispersal event itself.

Once arrived on an island, animals are likely to face-selective pressures that diverge from those experienced on the mainland in multiple aspects (see above §2), prompting adaptive, genetic changes in their personality and cognitive traits. Although studies on how personality traits evolve in response to environmental changes remain relatively rare [31], there is now evidence that changes in ecological factors such as food availability (e.g. [204]), predation pressure (e.g. [205]), parasite load (e.g. [206]) and habitat structure (e.g. [207]), all known to occur on islands, may drive personality evolution. In addition, personality traits seem a likely target of sexual selection (see [208,209] for an overview of ideas), whose strength and direction may vary among islands (e.g. [210–212]). Proof for the evolvability of personality comes from studies of the fitness gradient in wild populations, from artificial selection studies, and from analyses comparing populations or species (reviewed in [31,213]).

Probably because it is deemed key to the evolution of our own species, theories on why and when natural selection promotes high cognitive abilities abound (see e.g. [138] for a review). They can be pushed into two major schools. The ‘Social Intelligence Hypothesis’ postulates that cognition has evolved to meet the challenges of a complex social life, to be able to read the intentions of peers and manipulate their behaviour [214,215]. The ‘Ecological Intelligence Hypothesis' (EIH) states that other, non-social aspects of the environment have steered cognitive evolution: challenges associated with locating or manipulating food, finding shelter or avoiding predation, for instance [216]. Refining EIH, the widely cited ‘Cognitive Buffer Hypothesis’ [217] emphasizes the role of environmental variability and argues that cognition evolved as a means to buffer individuals against stochastic fluctuations in, for instance, food availability [123,218]. Instead, the ‘Expensive Tissue Hypothesis' (ETH) [136] argues how low or variable resource availability could select for reduced investment in costly brain tissue (and more performant gut tissue), which might come at the expense of cognitive ability. Artificial selection studies, selection gradient studies and comparative analyses have confirmed that cognition is indeed malleable through natural selection (see [138,219,220] for reviews). Interestingly, recent comparative genomic techniques found evidence for positive selection on genes associated with brain development in multiple lineages (e.g. dolphins: [221]; paper wasps: [222]; capuchin monkeys: [223], including our own [169,224]).

In short, insularization can affect personality and cognition for multiple reasons and through several pathways.

## 4. Challenges, opportunities and avenues for behavioural research on islands

If changes in personality and cognitive skills following insularization seem likely and important, then why have they not been studied more often? Clearly, behavioural traits tend to be highly plastic, do not fossilize well and are difficult to compare among species, all of which complicates evolutionary studies. However, these problems are not specific to island populations, and the recent spurt in animal cognition and personality research proves that they can be overcome. Actually, we believe that island–mainland or among-island comparisons constitute a very promising avenue for studying the micro-evolution of behaviour, just as they did for other traits and for the same reason: because they offer the opportunity to study recurrent phenotypic changes in relatively simple environments [5].

A possible explanation for the dearth of work on cognition and personality performed on insular systems could be a mismatch in study organisms. Studies of animal personality and cognition have traditionally used primates, other mammals, birds and fish as models [225,226]—species that are often not very abundant on islands—especially not on smaller, oceanic islands. Recently, however, techniques for measuring personality and cognitive capacity have been tailored to and successfully applied in other taxa, such as reptiles [227] and insects [228], that can be sampled in large numbers on even the smallest islands. With the right study organisms, it should be logistically possible to study how insularity affects personality and cognition.

Clearly, a number of quality criteria must be met. By definition, personality scores should be repeatable in time and across contexts, but this has rarely been assessed in island populations. Along the same line, cognitive scores should be carefully tested for repeatability within individuals, and consistency among individuals and across contexts. This requires recapturing the same individuals in sufficient numbers, which can be a major challenge in fast-moving animals. Obtaining robust behavioural measurements, preferably in a number of populations and species, is a necessary first step to establish whether there is, effectively, a behavioural component to the island syndrome.

Equipped with robust data on personality traits and cognitive abilities, hypotheses on how insularity incites changes in personality or cognition can then be put to the test (table 2). Several ideas on this matter can be formulated, but remain largely untested. For instance, inbreeding depression engenders individuals with maladaptive, under certain ecological contexts, personalities [141,161]. Predation intensity is considered a prime factor determining the relative fitness of different personalities (e.g. [205,229]), and an important driver of cognitive evolution (e.g. [230,231]). At least in some taxa, high population densities are expected to decrease aggressiveness and increase sociality in island dwellers (e.g. [232]), and in combination with low-resource availability, may constrain brain development and thus cognitive abilities [136]. By contrast, the unpredictable nature of island environments has been hypothesized to select for behavioural flexibility and, hence, superior cognitive abilities [103]. Inbreeding, predation relaxation, high population densities, increased intraspecific competition and environmental stochasticity are all examples of phenomena associated with, but not limited to, island environments. Therefore, studies on the behaviour of island dwellers will be of great value to our understanding of the evolution of personality and cognition, in general.

**Table 2.** Outstanding questions on the evolution of personality and cognition on islands.

**The master data**
Do island populations exhibit repeatable inter-individual differences in the way they behaviourally interact with their environment, and are these differences consistent across contexts?
Do populations (or communities) on islands exhibit the same range and relative frequencies of behavioural types as populations on the mainland, or on other islands?
Do animal populations on islands differ in cognitive skills from their counterparts on the mainland, or on other islands?
**The mechanisms of change**
What is the role of non-adaptive evolution (e.g. inbreeding, genetic drift, pleiotropy) in creating differences in cognition and personality between island and mainland populations?
What is the role of dispersal filtering in creating differences in cognition and personality between island and mainland populations? How long does this effect linger?
What is the role of phenotypic plasticity versus genetic adaptation in creating differences in cognition and personality between island and mainland populations?
**The drivers of change**
What is the effect of predator release on islands on personality traits? Are these effects general, or specific to a predatory context?
Does predator release on islands affect prey cognitive capacity? (How fast) do prey species lose their ability to recognize predators, to respond in adequate ways? Are these effects general, or specific to a predatory context?
How does reduced interspecific competition (and the possible resulting niche shift) on islands affect personality traits? Are these effects general, or specific to an interspecific context?
How do high population densities on islands affect personality?
How does low-resource availability or predictability affect personality traits, and cognitive capacity?
**Covariation with other characteristics**
Do changes in morphology (e.g. body size, shape and colour), physiology (e.g. brain size and digestive performance) or diet (type or breadth) observed in island populations concur with changes in personal or cognitive capacity? Are these changes adaptive or constrained?
Do changes in life history of the pace of life (fast to slow) on islands affect personality and cognition?
**Generality and relevance**
Are the magnitude and the direction of changes in personality and cognitive traits on islands consistent over taxonomic groups and island environments? If not, which factors are responsible?
How do personality and cognitive characteristics of island populations affect their vulnerability to alien species? How readily can island animals adjust personality and cognitive traits to cope with new challenges?

A logical next step would be to assess whether and how personality traits and cognitive traits covary among themselves, and with the morphological, physiological and life-history traits traditionally implicated in the ‘island syndrome’. This would allow testing outstanding hypotheses on how personality differences are maintained over time [233], on the existence and consistency of cognitive syndromes and styles [44,234], on the role of behaviour in the pace-of-life theory [140], and on behavioural consequences of correlational selection on physiological or morphological traits (e.g. [235–237]).

The above questions primarily relate to the eventual outcome of evolutionary trajectories, but islands also offer unique opportunities to learn about the nature of the trajectories themselves. By studying populations of varying age (colonization history), one could assess the importance of adaptive landscapes [238], genetic covariance matrices [239] and the prevalence of ‘evolutionary paths of least resistance’ [240] in the evolution of behaviour. Such analyses could also reveal reversals in the direction of evolution, e.g. when distinct phenotypes facilitate dispersal and settlement [183]. Finally, studying islands with different colonization histories could reveal information on the rate at which behavioural changes occur. Such knowledge is of fundamental biological interest, but in addition may be valuable in the context of the conservation of island and other isolated populations.

## Data Availability

This article has no additional data.
